# Role of a Novel Functional Variant in the *PPP2R1A* Promoter on the Regulation of PP2A-Aalpha and the Risk of Hepatocellular Carcinoma

**DOI:** 10.1371/journal.pone.0059574

**Published:** 2013-03-29

**Authors:** Hui-Feng Chen, Jian-Rong Mai, Jian-Xin Wan, Yan-fang Gao, Li-Na Lin, Song-Zi Wang, Yu-Xi Chen, Chen-Zi Zhang, Yu-Jing Zhang, Bin Xia, Kun Liao, Yu-Chun Lin, Zhong-Ning Lin

**Affiliations:** 1 School of Public Health, Xiamen University, Xiamen Fujian, PR China; 2 Guangdong Provincial Key Laboratory of Food, Nutrition and Health, School of Public Health, Sun Yat-sen University, Guangzhou, PR China; 3 Department of Laboratory Medicine, The First Affiliated Hospital, Sun Yat-sen University, Guangzhou, PR China; 4 Guangdong Prevention and Treatment Center for Occupational Disease, Guangzhou, PR China; Tongji Medical College, Huazhong University of Science and Technology, China

## Abstract

Previously, we identified the genetic variant −241 (−/G) (rs11453459) in the PP2A-Aα gene (*PPP2R1A*) promoter and demonstrated that this variant influences the DNA-binding affinity of nuclear factor-kappa B (NF-κB). In this study, we further confirmed that the transcriptional activity of *PPP2R1A* may be regulated by NF-κB through the functional genetic variant −241 (−/G). Moreover, we also demonstrated that the methylation status of CpG islands in the promoter of *PPP2R1A* influences the activity of this gene promoter. Few studies have examined the role of this −241 (−/G) variant in genetic or epigenetic regulation in hepatocellular carcinoma (HCC). To investigate whether this functional variant in the *PPP2R1A* promoter is associated with the risk of HCC and confirm the function of the −241 (−/G) variant in the HCC population, we conducted a case-control study involving 251 HCC cases and 252 cancer-free controls from a Han population in southern China. Compared with the −241 (−−) homozygote, the heterozygous −241 (−G) genotype (adjusted OR  = 0.32, 95% confidence interval (CI)  = 0.17–0.58, *P*<0.001) and the −241 (−G)/(GG) genotypes (adjusted OR  = 0.38, 95% CI  = 0.22–0.67, *P*  = 0.001) were both significantly associated with a reduced risk of HCC. Stratification analysis indicated that the protective role of −241 (−G) was more pronounced in individuals who were ≤ 40 years of age, female and HBV-negative. Our data suggest that the transcriptional activity of *PPP2R1A* is regulated by NF-κB through the −241 (−/G) variant and by the methylation of the promoter region. Moreover, the functional −241 (−/G) variant in the *PPP2R1A* promoter contributes to the decreased risk of HCC. These findings contribute novel information regarding the gene transcription of *PPP2R1A* regulated by the polymorphism and methylation in the promoter region through genetic and epigenetic mechanisms in hepatocarcinogenesis.

## Introduction

Protein phosphatase 2A (PP2A) is one of the major cellular serine-threonine phosphatases and is involved in many cellular processes, including metabolism, DNA replication, transcription, translation, cell cycle progression and apoptosis [1]. The PP2A holoenzyme consists of heterotrimeric forms generated by the association of a 36-kDa catalytic subunit C (PP2A-C) and a 65-kDa structural subunit A (PP2A-A) with over 15 different B subunits (PP2A-Bs) that influence substrate specificity and/or subcellular localization. Several PP2A subunits, such as B56α, B56γ, and PR72/130, have been implicated as tumor suppressors [2], [3]. Subunit A forms the scaffold of the holoenzyme and exists in two isoforms, Aα and Aβ, which share 86% amino acid identity and are encoded by the genes *PPP2R1A* and *PPP2R1B*, respectively. *PPP2R1A* is found on human chromosome 19q13.41, and *PPP2R1B* is found on 11q23.2 [4]. However, the Aβ isoform is much less abundant than the Aα isoform [5]. Genetically altered isoforms of both the Aα and Aβ subunits of PP2A have been reported in human melanomas as well as in breast and lung carcinomas [6].

We previously identified the genetic variant −241 (−/G) (rs11453459) in the 5′-flanking region of the *PPP2R1A* gene, located 241 bp upstream from the transcription start site (TSS, upstream from the TSS was defined as −1 nt). We determined that this −241 (−/G) variant influences DNA-protein interactions involving the transcription factor (TF) nuclear factor-kappa B (NF-κB), which may regulate the activity of the *PPP2R1A* promoter [7]. In our current study, a luciferase reporter assay further demonstrated that the functional genetic variant −241 (−/G) may influence the regulatory role of NF-κB in the transcriptional activity of *PPP2R1A* in human liver cell. However, the genetic variant alone cannot explain the diversity of gene regulatory mechanisms. DNA methylation, the best-known epigenetic marker, is also involved in the regulation of the promoter activities of targeted genes. The methylation of cytosine residues in the sequence 5′-cytosine-guanosine (CpG) found in gene promoter regions is the best-characterized epigenetic mechanism known, and it plays an important role in gene transcription, genome stability and genetic imprinting [8]. Aberrant hypermethylation of the CpG-rich promoter regions of tumor suppressor genes (TSGs) results in transcriptional silencing in a variety of solid tumors and blood cancers. *In vitro* and *in vivo* treatment with DNA methylation inhibitors, 5-Aza-2′-deoxycytidine (5-Aza-dC), has proven to be effective in restoring gene expression and normal patterns of differentiation and apoptosis in malignant cells [9]. However, the effects of *PPP2R1A* promoter methylation and genetic variant(s) in the *PPP2R1A* gene promoter on gene transcription have not been elucidated. In this study, to determine whether promoter methylation influences the transcription of *PPP2R1A*, we predicted the location of the CpG island in the *PPP2R1A* promoter region and examined the effect of promoter methylation and 5-Aza-dC treatment on the transcriptional activity of *PPP2R1A*. Moreover, we demonstrated that the methylation of deoxycytosine in the CpG islands of the *PPP2R1A* promoter is involved in regulating the transcriptional activity of *PPP2R1A,* and specifically, that PP2A-Aα expression was regulated by promoter hypomethylation. Taken together, these results suggest that the genetic variants and epigenetic status have been advanced as the possible mechanism in the gene transcriptional regulation of the *PPP2R1A* promoter.

Hepatocellular carcinoma (HCC) is one of the most common malignancies worldwide, though its incidence rate displays striking racial and geographic differences [10]. HCC is highly prevalent in China, especially in the south [11]. Genetic variation has been reported to influence the variable risk for HCC observed both within and across populations. Moreover, the environmental factors such as aflatoxin B1, HBV infection, HCV infection, alcohol consumption and tobacco smoking also increase the risk of the development of HCC [10], [11]. However, the genetic variants and epigenetics mechanisms for the regulation of gene transcription in the onset and progression of HCC pathogenesis remain unclear. Here, we hypothesized that this identified −241 (−/G) genetic variant in the *PPP2R1A* promoter may influence an individual’s susceptibility to hepatocarcinogenesis. To analyze the association of the *PPP2R1A* −241 (−/G) variant with risk of HCC and confirm the function of the −241 (−/G) variant in the HCC population, we genotyped the −241 (−/G) functional variant in a case-control study in a southern Chinese population. To our knowledge, we report here for the first time that individuals with the −241 G allele are at a reduced risk for HCC. Our results further suggest that the −241 (−/G) variant may be used as a biomarker for identifying the population subgroups that are the most susceptible to hepatocarcinogenesis.

## Materials and Methods

### Ethics Statement

This study was approved by the ethics committee of the School of Public Health, Sun Yat-sen University. The written informed consent was obtained from each subject, and all clinical investigation was conducted according to the principles expressed in the Declaration of Helsinki.

### Construction of Luciferase Reporter Plasmids Bearing the −241 (−/G) Variant

To study the effect of the previously identified −241 (−/G) variant on the transcriptional activity of the proximal promoter (−448 nt to +237 nt) region (downstream from the TSS was defined as +1 nt) [7], reporter plasmids containing the −241 (−/G) variant of the human *PPP2R1A* promoter were constructed by PCR from genomic DNA samples containing the native variant. A pair of upstream (−448XF6) and downstream (+237HR3) PCR primers flanked by restriction sites at the 5′-end were designed as previously described [7]. Two 685-bp fragments, bearing different −241 (−/G) alleles in the *PPP2R1A* promoter (2R1Ap), were separately cloned in the 5′-3′-orientation into the pGL3-Basic Luciferase Reporter Vector (Promega). The recombinant reporter constructs, designated as pGL3b-2R1Ap-241 (−) and pGL3b-2R1Ap-241G, were confirmed by sequencing.

### Methylation of Luciferase Reporter Plasmids Bearing the −241 (−/G) Variant

The prediction of putative CpG islands was performed using the CpG finder at the European Bioinformatics Institute (EMBL, http://www.ebi.ac.uk/) and the following parameters: CpG length >200 bp, G+C >50% and a “CpG value” of at least 0.6 [12].

Because the −241 (−/G) variant was located in the CpG-rich region, we then determined whether DNA methylation of the proximal promoter region could affect the transcriptional regulation of *PPP2R1A* through the −241 (−/G) variant. Ten micrograms of the pGL3b-2R1Ap-241(−), pGL3b-2R1Ap-241G constructs or the pGL3-Basic vector (pGL3b) were then CG methylated using 8 U of CpG methyltransferase (*M. Sss*I), 0.2 µl of *S*-adenosyl methionine (SAM) (New England Biolabs) and 4 µl of 10× reaction buffer in a final volume of 40 µl. The reactions were incubated at 37°C for 16 h, with subsequent inactivation of the enzyme at 65°C for 20 min. Mock-methylation reactions were also performed in the absence of *M. Sss*I and SAM. The methylated or mock-methylated constructs were purified using a QIAquick PCR purification kit (QIAGEN), and the methylation status of each construct was determined by comparing their digestion profiles using the *Hpa*II (methylation sensitive) and the *Msp*I (methylation insensitive) restriction enzymes (New England Biolabs).

### Transient Transfection and Luciferase Reporter Assays

The immortalized human normal hepatocyte L02 cell line was kindly provided by Dr. Shi-mei Zhuang (Sun Yat-sen University) [13]. For the luciferase reporter assays, L02 cells (1×10^4^) were plated in 96-multiwell plates and grown to 80–90% confluence. Transfection was conducted using the Lipofectamine™ 2000 Reagent (Life Technologies), according to the manufacturer’s instructions and our previous study [7], [14].

To examine the transcriptional activity of the *PPP2R1A* promoter bearing the −241(−/G) variant and analyze the role of NF-κB in the regulation of promoter activity of constructs with different alleles, cells were cotransfected with 50 ng of pRL-TK vector DNA (Promega) and 200 ng of either empty pGL3-Basic plasmid (used as a promoterless control) or reporter gene plasmids (pGL3b-2R1Ap-241(−) or −241G) harboring different allelotypes of the *PPP2R1A* promoter. As our previous study in nasopharyngeal carcinoma (NPC) cells [15], the human liver L02 cell models for the induction or inhibition of NF-κB activation were conducted in our current studies. After 4 h of transfection, cells that were transfected with the different plasmids above were treated with 25 ng/mL TNF-α (Sigma), as an inducer of NF-κB activation; 20 µM parthenolide (PN, Sigma), as an inhibitor of the NF-κB pathway; or pretreated with PN for 4 h then stimulated with TNF-α (PN+TNF-α) for another 24 h [15]. Negative control cells that transfected with variant reporter plasmids were maintained in fresh cell culture medium without treatment.

To further investigate the effects of methylation on the transcriptional activity of *PPP2R1A* promoter and to study the role the −241 (−/G) variant plays in the regulation of methylation on the transcription of *PPP2R1A*, the methylated or mock-methylated pGL3-Basic empty vector and 200 ng of the methylated or mock-methylated pGL3b-2R1Ap-241(−) or −241G constructs were cotransfected with 50 ng of pRL-TK into L02 cells. And the hepatocellular carcinoma HepG2 cell line was used to further confirm the *PPP2R1A* promoter activity with various methylation statuses measured by Dual Luciferase Assays. Co-transfection of the pRL-TK and the pGL3-Basic empty vectors was used as a negative control.

After incubation with transient transfection treatment for 24 h, the cells were harvested and assayed for firefly and *Renilla* luciferase activities with a Dual-Luciferase Reporter Assay System (Promega), as in our previous study [16]. All transfections were performed in triplicate and repeated at least thrice in independent experiments. Promoter activity was reported as relative light units (RLU) and calculated by defining the firefly activity of the empty pGL3-Basic vector as 1 RLU.

### Western Blot Analysis

To identify the relationship between the transcriptional regulation of the −241 (−/G) functional variant in the *PPP2R1A* promoter and the activation of NF-κB, nuclear proteins were extracted from L02 cells that were treated with TNF-α, PN, or PN+TNF-α. The extraction was performed using a Nuclear Extraction Kit (Active Motif) [7]. In total, 50 µg of nuclear proteins were used for each lane of loaded samples. The protein blots were blocked with 5% fat-free milk in TBS buffer for 1 h and then incubated for overnight at 4°C with antibody against NF-κB p65 (Santa Cruz Biotechnology Inc.) and β-actin antibody (Cell Signaling Technology) at a dilution of 1∶1000 and 1∶3000, respectively. The secondary antibody was anti-goat IgG or anti-mouse IgG (Santa Cruz Biotechnology Inc.) at a dilution of 1∶3000 or 1∶5000, respectively. The blots were detected using the enhanced chemiluminescence method (Pierce). The density of protein fragments was quantified using the GeneTools software package (version 3.03; SynGene).

### 5-Aza-2′-deoxycytidine Treatment

To confirm the effect of DNA methylation on the transcriptional activity of *PPP2R1A*, L02 cells cultured in 60-mm dishes were treated by the daily addition of cell culture medium supplemented with three different concentrations (2.5, 5.0 and 10.0 µM) of the DNA methylation inhibitor 5-Aza-2′-deoxycytidine (5-Aza-dC) (Sigma-Aldrich) for a total of 48 h. The assay was performed in triplicate at each of the 5-Aza-dC concentrations. Control cells were maintained in fresh cell culture medium without the addition of 5-Aza-dC. After treatment, cells were harvested to perform RNA extraction and real-time quantitative reverse transcription polymerase chain reaction (qRT-PCR).

### RNA Extraction and Real-time RT-PCR

Total RNA was extracted from L02 cells with and without 5-Aza-dC treatment using TRIzol^®^ Reagent (Life Technologies). Total RNA (500 ng) was reverse transcribed into single-strand complementary DNA (cDNA) using an oligo-dT primer and the PrimeScript™ RT Enzyme Mix Ι (SYBR® PrimeScript™ RT-PCR kit, TaKaRa). The quantification of the relative gene transcriptional level of *PPP2R1A* in the control cells and cells treated with 2.5–10 µM 5-Aza-dC, using *ACTB* (encoding β-actin) as an internal reference gene, was performed in triplicate for each sample using the ABI 7500 system and the SYBR-Green method. The following primers were used: *PPP2R1A*, forward, 5′-TCTGCATCAATGTGCTGTCT-3′, reverse, 5′-TTCACTCTGCAAGGTGCTGT-3′ and *ACTB*, forward, 5′-CACCAGGGCGTGATGGT-3′, reverse, 5′-CTCAAACATGATCTGGGTCAT-3′. The PCR reaction mixture consisted of 0.1 µmol/L of each primer, 1× SYBR® Premix EX Taq™ (Perfect Real Time) premix reagent (TaKaRa) and 50 ng of cDNA to a final volume of 20 µl. Cycling conditions consisted of 95°C for 30 s followed by 40 cycles at 95°C for 5 s and 60°C for 34 s. The averaged cycle threshold (C_T_) values for each reaction derived from the target gene, as determined using the ABI 7500 system software, were normalized to the levels of *ACTB*. The ΔC_T_ was then calculated by subtracting the C_T_ of *ACTB* from the C_T_ of *PPP2R1A*. The ΔΔC_T_ was calculated by subtracting the ΔC_T_ of the reference sample from the ΔC_T_ of each sample. The fold change was determined using the equation 2^−ΔΔCT^.

### Population Study Subjects

To further confirm the function of the −241 (−/G) variant in the population and investigate the association between this variant and HCC risk, we performed a case-control study in a southern Chinese population. The study subjects consisted of 251 HCC case patients and 252 cancer-free controls recruited from The First Affiliated Hospital, Sun Yat-sen University, between January 2008 and October 2011. All subjects in the study were unrelated ethnic Han Chinese confirmed by ID cards and “Household Register books”, which serve as identification in China as described in our previous study [16]. The individuals were living in Guangzhou and its surrounding regions (Guangdong Province, China) in southern China, where HCC is prevalent and HBV infection has been shown to be the main attributable risk factor [17], [18]. All cases were diagnosed with histopathologically confirmed HCC. Control subjects were health checkup examinees without a history of cancer who were recruited in the same period. The exclusion criteria included previous cancer, metastasized cancer arising from a different or unknown origin and previous radiotherapy or chemotherapy. All participants were negative for antibodies to the hepatitis C virus, hepatitis D virus or human immunodeficiency virus. In our present study, the HBV infection was identified in 88.4% of cases and in 12.7% of controls, respectively. Because genotype frequencies can vary among ethnic groups, only southern Han Chinese were included in this analysis [7]. We used a short questionnaire to obtain demographic and risk factor information and frequency matched the controls to the cases by age (±5 years) and sex. After physical examination, 5 ml of blood was drawn, labeled, and delivered to the lab for subsequent genomic DNA extraction, as in our previous study [16].

### Genotyping Analysis

Genomic DNA was extracted from the blood samples of all study subjects. An allelic discrimination method employing allele-specific ﬂuorogenic probes was used for genotyping along with the ABI PRISM 7500 Sequence Detection System (Life Technologies) [19]. Both the primers and the probes were designed and synthesized by Applied Biosystems. Polymerase chain reaction (PCR) was performed in 25 µl reaction mixtures under the following conditions: an initial melting step at 95°C for 10 min followed by 40 cycles of 92°C for 15 s and 60°C for 1 min. A multicomponent algorithm was used to calculate the distinct signal contributions of each allele from the ﬂuorescence measurements for each sample using the ABI 7500 real-time PCR system. To validate the genotyping results, approximately 10% of the PCR products were randomly selected and confirmed by direct sequencing, while another 50 samples (10%) were randomly selected for confirmation by repeated genotyping. All results were 100% concordant.

### Statistical Analysis

Either Student’s *t-*test for two groups or an ANOVA with an *S-N-K* test for multiple groups was used to examine the differences in the expression of the luciferase reporter gene. The *χ* ^2^ test was used to evaluate the differences in the frequency distributions between cases and controls of selected demographic variables and the known risk factors of HCC, such as HBV infection, as well as of each allele and genotype of the −241 (−/G) polymorphism. The Hardy-Weinberg equilibrium of the genotype distribution of the controls was tested by a *χ* ^2^ goodness-of-fit test to compare the expected genotype frequencies with the observed genotype frequencies (p^2^+2pq+q^2^ = 1) in cancer-free controls. The association between the genotype and the risk of HCC, as measured by the odds ratio (OR) and its corresponding 95% confidence interval (CI), was estimated using an unconditional logistic regression model with adjustment for age, sex and HBV infection. In the stratification analysis, we assessed the effects of the *PPP2R1A* genotypes in each subgroup and the selected variables on cancer risk. The statistical power was calculated using PS Software [20]. All tests of statistical significance were two-sided (*P*<0.05) and were performed using the SPSS statistical software package (version 16.0).

## Results

### The Transcriptional Activity of the *PPP2R1A* Promoter is Regulated by NF-κB through the −241 (−/G) Variant

Bioinformatics analysis of the *PPP2R1A* promoter revealed a putative binding site for NF-κB located in the potential functional variant −241 (−/G) identified in our previous study [7]. Therefore, we conducted experiments to determine the effect of the genetic variant on the regulation of *PPP2R1A* promoter activity through the transcription factor NF-κB.

**Figure 1:**
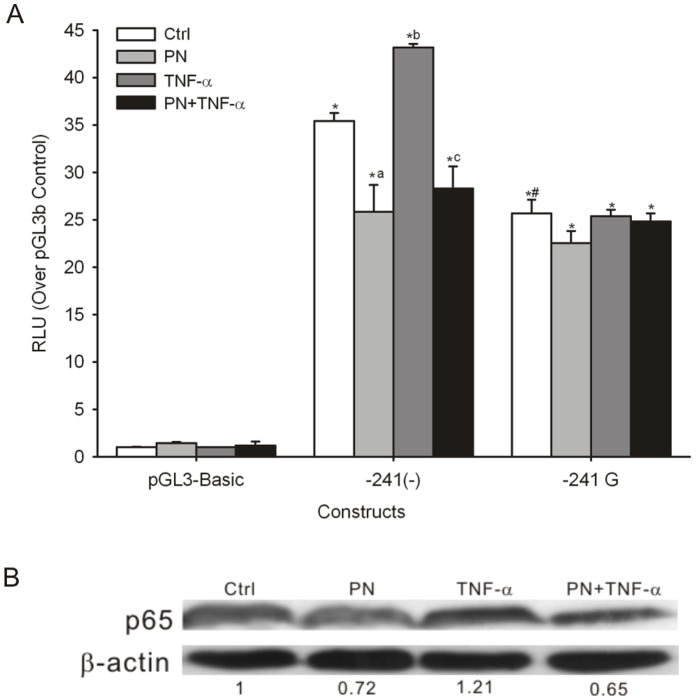
Functional analysis of the−241 (−/G) variant in the *PPP2R1A* proximal promoter. **A.** Transcriptional activity of fragments bearing the *PPP2R1A* proximal promoter with variant allelotype of −241 (−) or −241 G was measured by Dual Luciferase Assays in immortalized human normal hepatocyte L02 cells. The results are expressed as fold increases in RLU over the empty pGL3-Basic vector (Ctrl). Compared with the basal activity of Ctrl, the promoter activity of the pGL3b-2R1Ap-241(−) or −241G with or without treatment was higher (∗ *P*<0.05). Compared with pGL3b-2R1Ap-241(−) construct, the construct with −241 G allele had lower activity (**^#^** *P<*0.05). Compared with the Ctrl of pGL3b-2R1Ap-241(−), the promoter activity of constructs containing −241 (−) was significantly decreased when treated with parthenolide (PN) (**^a^** *P*<0.05), and was significantly increased after TNF-α induction (**^b^** *P*<0.05). Compared with the TNF-α treatment alone, the promoter activity was decreased after treatment with PN+TNF-α in constructs carrying the −241 (−) allele (**^c^** *P*<0.05). However, there was not changed of the promoter activity in the constructs with the −241 G allele among the Ctrl and the different treatment groups (*P*>0.05). **B.** Immunoblot characterization of the nuclear NF-κB p65 translocation after treatment with NF-κB activator or inhibitor in L02 cells. The expression of p65 was inhibited 28% after treatment with PN, while the expression of p65 was significantly increased by 21% after treatment with TNF-α. When pretreated with PN for 4 h and stimulated with TNF- for another 24 h (PN+TNF-α), the expression of p65 was still decreased by 35% in L02 cells (*P*<0.05). The numbers under the graph represent the fold of relative expression level of nuclear NF-κB p65 (Ctrl group level set as 1.0). The results represent the mean ± SD of three independent experiments.

To assess the impact of the −241 (−/G) variant on the transcriptional activity of the *PPP2R1A* proximal promoter, we generated the pGL3b-2R1Ap-241(−) or −241G constructs because the NF-κB binding site is located in this core promoter region. The recombinant plasmids were transfected into L02 cells. The empty vector (pGL3-Basic) and a *Renilla* construct (pRL-TK) were used as controls (pGL3b). As shown in Fig. 1A, the two *PPP2R1A* proximal promoter constructs generated higher levels of luciferase expression than the empty vector control (*P*<0.05). These results suggest that the proximal region in the 5′-flanking of *PPP2R1A* has the higher transcriptional activity, consistent with the core promoter identified in our previous study [7]. The two constructs also differed from each other in luciferase expression. The construct containing the −241 (−) allele displayed the higher transcriptional activity than the construct containing the −241 G allele (*P*<0.05). These data indicate that the different allelotypes, −241 (−) or −241 G, have significant effects on *PPP2R1A* promoter transcription activity in human liver cells.

To confirm the regulatory role of the −241 (−/G) genetic variant in the promoter activity of *PPP2R1A* via NF-κB regulatory mechanism, we examined the reporter gene activity of pGL3b-2R1Ap-241(−) and −241G after treatment with an NF-κB activator or inhibitor in L02 cells. As shown in Fig. 1A, the control experiment demonstrated that treatment with TNF-α or PN did not affect the basal luciferase activity of the pGL3-Basic vector. Compared with the pGL3b-2R1Ap-241(−) control group, the level of reporter gene activity of the construct containing the −241 (−) allele was 1.22-fold higher after TNF-α induction (*P*<0.05). However, reporter activity was not increased in the construct containing the −241 G allele (*P*>0.05) (Fig. 1A). Next, treatment with PN, an inhibitor of the NF-κB pathway, resulted in reporter activity being significantly decreased 27% in constructs containing the −241 (−) allele (*P*<0.05). However, the reporter activity of the construct containing the −241 G allele was not significantly suppressed after PN treatment (*P*>0.05) (Fig. 1A). Then, to confirm the selectivity and specificity of the regulatory role of NF-κB, L02 cells were treated with PN+TNF-α. The reporter activity was decreased after PN+TNF-α treatment in the construct carrying the −241 (−) allele (*P*<0.05) but was not changed in the construct with the −241 G allele (*P*>0.05), compared with TNF-α or PN treatment alone (Fig. 1A). The results showed that the TNF-α-induced NF-κB activation, the PN-induced NF-κB inhibition affect the promoter activity of *PPP2R1A* harboring with the −241 (−) allele but not with the −241 G variant. It was indicated that the NF-κB mechanism may involve in the regulation of *PPP2R1A* transcription via the −241 (−/G) genetic variant in liver cell. Furthermore, we validated the effects of PN or TNF-α treatment on the activated expression of NF-κB p65, an obligate ingredient of the NF-κB heterodimer, in L02 cells nuclear extracts (Fig. 1B). It was found that PN treatment could inhibit the expression of NF-κB p65 by 28%, while TNF-α significantly increased the expression of p65 by 21%. In the PN+TNF-α group, the expression of NF-κB p65 was still decreased by 35% (*P*<0.05) (Fig. 1B). These results suggest that the activation intervention of NF-κB consistent with the reporter gene assay. The *PPP2R1A* gene promoter regulated by the −241 (−/G) genetic polymorphism and the target of NF-κB were confirmed by the human liver cell models. Taken together, these results further confirmed the predicted NF-κB binding site in cellular functional assay and indicated that activation or inhibition of NF-κB, involved in the transcriptional regulation of *PPP2R1A* and the −241 (−/G) variant, may play an important role in the regulation of *PPP2R1A* promoter function through NF-κB transcriptional activity in human hepatocytes.

### Methylation of the *PPP2R1A* Promoter Suppresses its Transcriptional Activity

**Figure 2:**
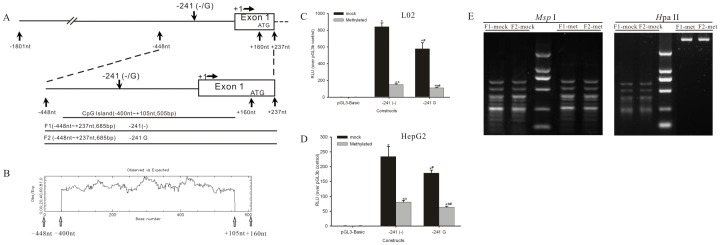
Effect of plasmid DNA methylation on *PPP2R1A* promoter activity. **A.** The *PPP2R1A* genomic reference sequence (with GenBank accession no. AC 010320) was used with the transcription start site (TSS) set as nucleotide +1. The variant identified in the current study, −241 (−/G) (rs11453459), is located in the *PPP2R1A* promoter region (−1801 nt to +237 nt). The two fragments, designated as 2R1Ap-Fs, including F1 ((−)^−241^) and F2 ((G) ^−241^), are located in the *PPP2R1A* proximal promoter (−448 nt to +237 nt, 685 bp). **B.** The CpG islands of the 5′-flanking region of *PPP2R1A*. The region from −400 nt to +105 nt was identified as a CpG island using the CpG finder at EMBL (http://www.ebi.ac.uk/) and the following parameters: CpG length >200 bp, G+C >50% and a “CpG value” of at least 0.6. **C.** The *PPP2R1A* promoter activity with various methylation statuses was measured by Dual Luciferase Assays in L02 cells. The promoter activity in the luciferase reporter assays decreased significantly following plasmid DNA methylation. Compared with the basal activity of the pGL3-Basic control (Ctrl, which the relative luciferase activity was given a value of 1 RLU), the promoter activity of all constructs was increased by statistically significant levels (∗ *P*<0.01). Compared with the luciferase activity of unmethylated pGL3b-2R1Ap-241(−) construct or −241G construct, the promoter activity of the methylated −241(−) construct or −241G construct was decreased by statistically significant levels (^a^ *P*<0.01 and ^b^ *P*<0.01, respectively). Compared with the luciferase activity of the methylated F1 construct with −241 (−), the promoter activity of the methylated F2 construct with −241 G was decreased by statistically significant levels (^#^ *P*<0.01). **D.** The *PPP2R1A* promoter activity with methylation was measured in HepG2 cells. The promoter activity in the luciferase reporter assays decreased significantly following plasmid DNA methylation. Compared with the basal activity of Ctrl (given as 1 RLU), the promoter activity of all constructs was in significant higher levels (∗ *P*<0.01). Compared with the activity of unmethylated pGL3b-2R1Ap-241(−) construct or −241G construct, the promoter activity of the methylated −241(−) construct (^a^ *P*<0.01) or −241G construct (^b^ *P*<0.01) was decreased significantly respectively. Compared with the luciferase activity of the unmethylated or methylated pGL3b-2R1Ap-241(−) construct, the promoter activity of the −241G construct was decreased by statistically significant levels (^#^ *P*<0.01). **E.** Methylation status was confirmed by comparing the plasmid’s digestion profile with *Hpa*II (methylation sensitive) and *Msp*I (methylation insensitive) restriction enzyme. A series of *PPP2R1A* luciferase reporter constructs and its empty vectors were treated with or without *M. Sss*I and its substrate SAM. The results represent the mean ± SD of three independent experiments.

The regulation of gene expression is a complex process that is achieved through the action of transcription factors (TFs) and epigenetic regulatory mechanisms including DNA methylation. In addition to the effect of the −241 (−/G) functional genetic variant on the regulatory role of NF-κB, we hypothesized that DNA methylation in the *PPP2R1A* promoter may alter its transcriptional activity. The putative CpG island (−400 nt to +105 nt) in the *PPP2R1A* proximal promoter region (−448 nt to +160 nt) was predicted using the CpG finder at EMBL (Fig. 2A, Fig. 2B).

We then conducted luciferase reporter assays to determine the effect of methylation on the transcriptional activity of the *PPP2R1A* promoter. The pGL3b-2R1Ap-241(−) or −241G constructs containing different −241 (−/G) alleles, along with the pGL3-Basic vector, were methylated using *M. Sss*I and its substrate SAM. As shown in Fig. 2C, the reporter gene activity of the methylated plasmid was significantly decreased by 82% for the pGL3b-2R1Ap-241(−) allele construct (*P*<0.01) and 81% for pGL3b-2R1Ap-241G construct (*P*<0.01) when compared with the luciferase activity of unmethylated counterpart constructs respectively in L02 cells (Fig. 2C). And compared with the luciferase activity of the methylated F1 construct with −241(−), the promoter activity of the methylated F2 construct with −241G was decreased by statistically significant levels (*P*<0.01). The control experiment demonstrated that methylation did not affect the basal luciferase activity of the pGL3-Basic vector. The transfection efficiency was not affected by methylation of the plasmids because cells cotransfected with either methylated or unmethylated pRL-TK vectors exhibited approximately the same *Renilla* luciferase activities. To confirm the influence of methylation on the promoter activity of *PPP2R1A*, we further performed the luciferase reporter assay in HepG2 cell lines (Fig. 2D). Compared with the luciferase activity of respective unmethylated pGL3b-2R1Ap-241(−) construct or −241G construct, the promoter activity of the methylated pGL3b-2R1Ap-241(−) construct or −241G construct was decreased by statistically significant levels (*P*<0.01) (Fig. 2D). It shows that the regulatory function of methylation on the *PPP2R1A* promoter activity in HepG2 cells was similar to that observed in L02 cells (Fig. 2C, Fig. 2D). These results suggest that the *PPP2R1A* promoter activity displayed by the methylated construct was significantly suppressed compared with the mock-methylated construct. The methylation status of the plasmids here was confirmed by the plasmid digestion profiles as created with *Hpa*II and *Msp*I restriction enzymes. The *Hpa*II will not cut sites that have been methylated by *Sss*I methyltransferase, while the *Msp*I will cut sites, regardless of methylation status by *Sss*I methyltransferase (Fig. 2E). These results indicate that the methylation of the *PPP2R1A* promoter plays an important role in the regulation of *PPP2R1A* transcription activity.

Moreover, as shown in Fig. 2C and Fig. 2D, the methylation of the plasmid bearing the different −241 (−/G) variants still displayed higher promoter activity compared with pGL3-Basic vector (*P*<0.05), suggesting that the transcriptional regulation of *PPP2R1A* is dependent other factors than the epigenetic mechanisms of DNA methylation. These results also demonstrate that the methylated construct harboring the −241 (−) allele displayed higher promoter activity than the methylated construct containing the −241 G allele (*P*<0.05). These results further indicate that the effect of DNA methylation on the regulation of *PPP2R1A* promoter activity may partially correlate to the genetic variation present in the promoter region of this gene.

### Demethylation of the Promoter Enhances the Expression of *PPP2R1A*

**Figure 3:**
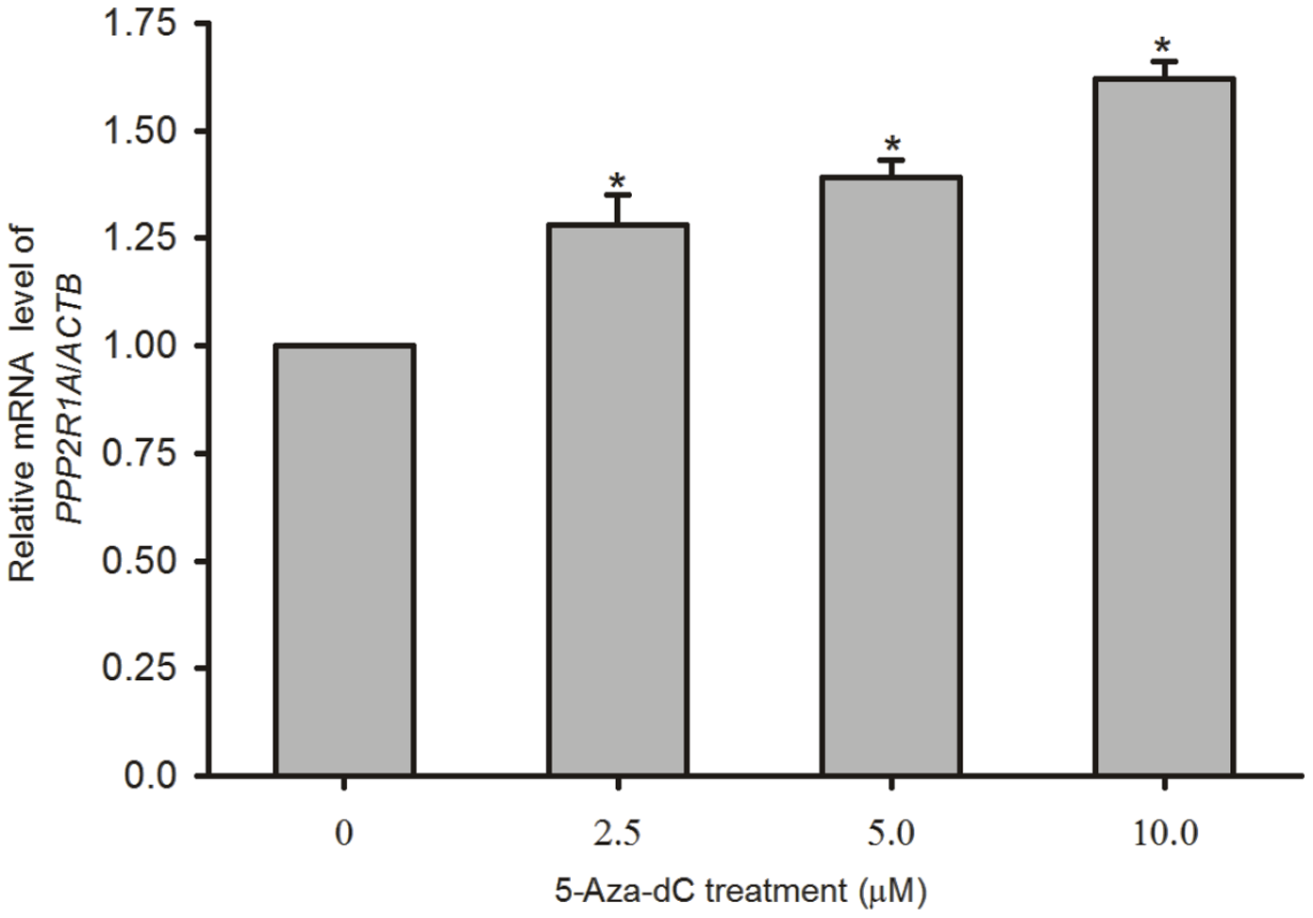
Treatment with 5-Aza-dC upregulates the transcription of *PPP2R1A* in L02 cells. RNA was extracted from immortalized human normal hepatocyte L02 cells treated with or without 5-Aza-2′-deoxycytidine (5-Aza-dC), a DNA methylation inhibitor, for 48 hours at the indicated concentrations (2.5, 5.0 and 10.0 µM), and the *PPP2R1A* transcripts in the control group and the 5-Aza-dC treated group were quantified using real-time RT-PCR (normalized against *ACTB*). The results were normalized against the untreated control (Ctrl), which was given a value of as 1.0. Compared with the Ctrl, the levels of *PPP2R1A* mRNA in the 5-Aza-dC treated groups were increased by statistically significant levels (∗ *P*<0.05). The level of *PPP2R1A* mRNA in the 10.0 µM 5-Aza-dC treatment group was significantly higher than the mRNA level in the 2.5 µM 5-Aza-dC treatment group (**^#^** *P<*0.05). The results represent the mean ± SD of at least three independent experiments performed in duplicate.

The previous results demonstrated that the hypermethylation of *PPP2R1A* promoter could inhibit transcription activity. To further confirm the effects of DNA demethylation on the transcriptional regulation of *PPP2R1A* in cells, we performed demethylation studies using 5-Aza-dC. As shown in Fig. 3, the mRNA levels of *PPP2R1A* increased in a dose-dependent manner in L02 cells after 5-Aza-dC treatment. The increase, when using 5 µM and 10 µM of 5-Aza-dC, reached statistically significant values (*P*<0.05). A 1.39-fold increase in the transcription of *PPP2R1A* was observed in cells treated with 5 µM of 5-Aza-dC. Compared with the control cells, 10 µM of 5-Aza-dC increased the expression of *PPP2R1A* by 0.62-fold in L02 cells. These results indicate that the increase in *PPP2R1A* transcription is associated with the hypomethylation of the *PPP2R1A* promoter and that the methylation status plays a partial role in the regulation of *PPP2R1A* promoter activity. Taken together and based on the role of genetic polymorphism in the regulation of *PPP2R1A* transcriptional activity in the aforementioned results, this study further confirms the methylation epigenetic mechanisms may also be involved in a regulatory role. These results suggest the existence of an interaction between genetic-epigenetic mechanisms in the transcription of *PPP2R1A* that is regulated by the polymorphism and methylation in this gene promoter region in human hepatocytes. However, to determine whether this functional −241 (−/G) variant is involved in hepatocarcinogenesis susceptibility required further population studies.

### The Distributions of *PPP2R1A* −241 (−/G) Genotypes and the Risk of HCC

We have identified a potential functional −241 (−/G) variant and confirmed its regulatory role in hepatocytes. To test the hypothesis that the −241 (−/G) (rs11453459) variant in the *PPP2R1A* promoter is associated with the HCC risk, we conducted a case-control study in a southern Chinese population. The distribution of demographic characteristics for the 251 HCC cases and 252 cancer-free controls are listed in Table 1. The mean age was 44.2 years for the cases and 43.0 years for the control subjects. Overall, the differences in the distributions of age and sex between the cases and controls were not statistically significant (*P*  = 0.18 and *P*  = 0.15, respectively), suggesting that the matching based on these two variables was adequate. However, the incidence of HBV infection in the HCC group (88.4%) was much higher than in the control group (12.7%) (*P*<0.001), implying that HBV infection was a critical risk factor for the development of HCC in our study population (Table 1).

**Table 1:** Frequency distributions of selected variables in HCC case and control subjects.

Variables	Case (n = 251), n (%)	Control (n = 252), n (%)	*P* a
Age (mean ± SD) (years)	44.17±9.23	42.96±10.68	0.176
Age (years)			0.18
≤ 40	84 (33.5)	99 (39.3)	
> 40	167 (66.5)	153 (60.7)	
Sex			0.15
Male	217 (86.5)	206 (81.7)	
Female	34 (13.5)	46 (18.3)	
HBV^b^			< 0.001
+	222 (88.4)	32 (12.7)	
−	29 (11.6)	220 (87.3)	

a *P* value for a two-sided *χ* ^2^ test.

b +/− : presence or absence of HBV infection.

First, we chose to investigate the variant in the Cantonese Han population by genotyping. The allele frequencies and genotype distributions of the *PPP2R1A* −241 (−/G) variant among the cases and controls are summarized in Table 2. The observed genotype frequencies of this variant were in agreement with Hardy-Weinberg equilibrium in the control subjects (*P*>0.05), indicating that the genotype distributions were not biased by our control selection. We observed genotype frequencies of 64.9% (− −), 27.9% (−G) and 7.2% (GG) in the HCC group, and 41.7% (− −), 48.8% (−G) and 9.5% (GG) in the control group, respectively. The genotype distribution of the −241 (−/G) variant in the HCC group was significantly different from the control group (*χ* ^2^ = 27.96, *P*<0.001). Moreover, the variant G allele frequency was 21.1% for the cases and 33.9% for the controls, which was also statistically significant (*χ* ^2^ = 20.69, *P*<0.001). These findings strongly suggest that the functional −241 (−/G) variant may be associated with the risk of HCC.

**Table 2:** Distribution of *PPP2R1A* gene promoter genotypes and their association with the risk of HCC.

Genotypes/Alleles	Case (n = 251), n (%)	Control (n = 252), n (%)^a^	Adjusted OR (95% CI)^b^	*P* c
Total no. of subjects	251	252		
Total no. of alleles	502	504		
−241 (−/G) (rs11453459)				
(− −)	163 (64.9)	105 (41.7)	1.00 (ref.)	
(−G)	70 (27.9)	123 (48.8)	0.32 (0.17–0.58)	<0.001
(GG)	18 (7.2)	24 (9.5)	0.79 (0.29–2.16)	0.65
[(−G)+(GG)]	88 (35.1)	147 (58.3)	0.38 (0.22–0.67)	0.001
Alleles				
−	396 (78.9)	333 (66.1)	1.00 (ref.)	
G	106 (21.1)	171 (33.9)	0.52 (0.39–0.69)	<0.001

a All observed genotype frequencies among the control subjects were in agreement with Hardy-Weinberg equilibrium (p^2^+2pq+q^2^ = 1) (for −241 (−/G), *χ* ^2^ = 0.335, *P* = 0.568 among the southern Chinese).

b Adjusted in a logistic regression model that included age, sex and HBV status. OR, odds ratio; CI, confidence interval.

c *P* values for the test of the multiplicative interaction between the *PPP2R1A* promoter −241 (−/G) variant and selected variables on cancer risk were calculated using unconditional logistic regression models.

We used logistic regression analysis to evaluate the association between the −241 (−/G) variant and the risk of HCC in this southern Chinese population. Compared with the −241 (− −) homozygous wild-type genotype, heterozygous carriers of the −241 (−G) variant genotype had a decreased risk of HCC (OR  = 0.32, 95% CI  = 0.17–0.58, *P*<0.001), while homozygous carriers of the −241 (GG) genotype displayed no significant differences between cases and controls (OR  = 0.79, 95% CI  = 0.29–2.16, *P*  = 0.65). A similar trend of decreased risk for HCC was detected for −241 (−G)/(GG) genotypes (OR  = 0.38, 95% CI  = 0.22–0.67, *P*  = 0.001). These data indicate that the *PPP2R1A* −241 G allele may be associated with a lower risk of HCC. In addition, these results further confirm the functional importance of the −241 (−/G) variant in this population study.

### Stratification Analysis of the *PPP2R1A* −241 (−/G) Variant and the Risk of HCC

HCC is known for its genetic heterogeneity. Thus, we performed a stratification analysis on the associations between the −241 (−/G) variant of *PPP2R1A* and the risk of HCC by subgroups according to age, sex and HBV infection status. As shown in Table 3, we found that the association between the −241 (−G) genotype and the decreased risk of HCC was more pronounced in subjects aged ≤ 40 years (adjusted OR  = 0.22, 95% CI  = 0.07–0.73 for the −241 (−G) genotype,) when compared with the homozygous −241 (− −) genotype. Moreover, the risk associated with the combined variant genotypes −241 (−G)/(GG) was also more pronounced in subjects aged ≤ 40 years (OR  = 0.21, 95% CI  = 0.07–0.63). We also observed a significant protective role of the −241 (−G) genotype and −241 (−G)/(GG) genotypes in female subjects (adjusted OR  = 0.11, 95% CI  = 0.03–0.48 for the −241 (−G) genotype, and OR  = 0.13, 95% CI  = 0.03–0.54 for the −241 (−G)/(GG) genotypes) and in HBV-negative subjects (adjusted OR  = 0.23, 95% CI  = 0.08–0.64 for the −241 (−G) genotype, and OR  = 0.29, 95% CI  = 0.12–0.73 for the −241 (−G)/(GG) genotypes). However, no interactions were observed between age, gender, or HBV infection and the variant −241 (−/G) genotypes.

**Table 3:** Stratified analyses between *PPP2R1A* promoter variant −241 (−/G) (rs11453459) genotypes and the risk of HCC.

	Case, n (%)	Control,n (%)	Adjusted OR(95% CI) a	*P* value
**Ages(years)**				
≤ 40				
(− −)	56 (66.7)	38 (38.4)	1.00 (ref.)	
(−G)	22 (26.2)	47 (47.5)	0.22 (0.07–0.73)	0.013
(GG)	6 (7.1)	14 (14.1)	0.16 (0.03–0.91)	0.039
[(−G)+(GG)]	28 (33.3)	61 (61.6)	0.21(0.07–0.63)	0.005
**> 40**				
(− −)	107 (64.1)	67 (43.8)	1.00 (ref.)	
(−G)	48 (28.7)	76 (49.7)	0.34 (0.16–0.72)	0.004
(GG)	12 (7.2)	10 (6.5)	1.94 (0.56–6.66)	0.295
[(−G)+(GG)]	60 (35.9)	86 (56.2)	0.47 (0.24–0.93)	0.03
**Sex**				
**Male**				
(− −)	143 (65.9)	100 (48.5)	1.00 (ref.)	
(−G)	59 (27.2)	85 (41.3)	0.36 (0.18–0.71)	0.004
(GG)	15 (6.9)	21 (10.2)	0.82 (0.27–2.50)	0.730
[(−G)+(GG)]	74 (34.1)	106 (51.5)	0.42 (0.22–0.80)	0.009
**Female**				
(− −)	20 (58.8)	5 (10.9)	1.00 (ref.)	
(−G)	11 (32.4)	38 (82.6)	0.11 (0.03–0.48)	0.003
(GG)	3 (8.8)	3 (6.5)	0.33 (0.03–3.23)	0.342
[(−G)+(GG)]	14 (41.2)	41 (89.1)	0.13 (0.03–0.54)	0.005
**HBV status**				
**+**				
(− −)	143 (64.4)	13 (40.6)	1.00 (ref.)	
(−G)	64 (28.8)	18 (56.2)	0.35 (0.16–0.76)	0.008
(GG)	15 (6.8)	1 (3.1)	1.42 (0.17–12.02)	0.750
[(−G)+(GG)]	79 (35.6)	19 (59.4)	0.40 (0.19–0.87)	0.021
**−**				
(− −)	20 (69.0)	92 (41.8)	1.00 (ref.)	
(−G)	6 (20.7)	105 (47.7)	0.23 (0.08–0.64)	0.005
(GG)	3 (10.3)	23 (10.5)	0.58 (0.16–2.14)	0.412
[(−G)+(GG)]	9 (31.0)	128 (58.2)	0.29 (0.12–0.73)	0.008

a ORs were adjusted for age, sex and HBV infection status using a logistic regression model. OR, odds ratio; CI, confidence interval.

## Discussion

As our results demonstrate, for the first time at the cellular level, the functional −241 (−/G) variant in the promoter may regulate the transcriptional activity of *PPP2R1A* by modulating the transcription factor NF-κB in human hepatocytes. In addition, the transcription of *PPP2R1A* is also regulated by the methylation of the promoter, which correlated with the genetic variant in the regulatory region. Furthermore, we also demonstrated that the functional −241 (−/G) variant in the *PPP2R1A* promoter contributes to the decreased risk of HCC in southern Han Chinese.

Single nucleotide polymorphisms (SNPs) are the most common form of human genetic variation, and the regulatory SNPs (rSNPs) located in the regulatory regions of human genes can significantly influence to the cellular level of mRNA transcripts produced and the individual susceptibilities to cancer [21], [22], [23]. Existing evidence has demonstrated that certain regulatory variants found in the promoter regions of genes can disturb the binding of transcription factors (TFs), thus affecting target gene expression and the associated risk of cancer [17], [24], [25], [26]. PP2A accounts for the majority of the serine-threonine phosphatase activity in mammalian cells and is involved in the regulation of numerous signaling pathways [27]. Moreover, several PP2A subunits have been implicated as tumor suppressors in recent studies [2], [3], [28]. Inactivating somatic mutations occurring in both of the structural subunit genes of PP2A, *PPP2R1A* and *PPP2R1B* have been identified. These genetic variants in the *PPP2R1A* coding region have been consistently associated with several cancers, including breast cancer, lung cancer, melanoma, and colon cancer [6], [29], [30], [31]. At least four PP2A-Aα isoform somatic mutants have been identified in human tumors, including a glutamic acid-to-aspartic acid (E64D) substitution in lung carcinoma, a glutamic acid-to-glycine (E64G) substitution in breast carcinoma, an arginine-to-tryptophan (R418W) substitution in malignant melanoma and a frame-shift mutation at nucleotide position 652 in a breast carcinoma [6]. Chen et al. reported that cancer-associated Aα mutations contribute to cancer development by inducing functional haploinsufficiency, disturbing PP2A holoenzyme composition and altering the enzymatic activity of PP2A [32]. The loss of *PPP2R1A* protein expression has also been observed in breast cancer [33]. However, the specific functional genetic variants located in the *PPP2R1A* promoter and its association with the risk of HCC has not yet been defined.

In our previous study, we designated a proximal region of 685 bp (−448 nt to +237 nt) as the core promoter of *PPP2R1A* because it displayed the highest transcriptional activity in luciferase assays [7]. In the current study, we constructed proximal promoter fragments mirroring the *PPP2R1A* allelotypes, including either the −241 (−) or the −241 G allele, to determine transcriptional activity. Promoter activity assays revealed that the allelotype −241 (−) exhibited a higher promoter activity. The other 5′-flanking fragment bearing −241 G displayed a lower level of promoter activity. Our results suggest that different allelotypes composed of the −241 (−/G) variant influence the transcriptional regulation of the *PPP2R1A* promoter. A recent report demonstrated that the −241 (−/G) variant was located in the *PPP2R1A* promoter region, which is regulated by the binding of TFs such as ETS-1, CREB, AP-2α and Sp1 [34]. In our previous study, we also reported that the functional −241 (−/G) variant may influence its binding affinity to the transcription factor NF-κB [7]. Moreover, it was confirmed that the activity of the *PPP2R1A* promoter was affected by −241 (−/G) variants via NF-κB activation in our current study.

The DNA methylation of promoter regions is one of the major regulatory mechanisms controlling gene transcription and expression in various biological processes [35]. The term DNA methylation usually refers to the post-synthetic methylation of deoxycytosine (dC) residues at the 5′ position to form deoxymethylcytosine (dmC). Frequently, both the core promoter and transcription initiation site are included within CpG islands, and gene expression is completely repressed when these islands become hypermethylated [36], [37]. DNA methylation is essential for mammalian development and health, and aberrant DNA methylation contributes to the pathogenesis and progression of disease, including cancer. A few studies have investigated the effect of methylation on the expression of the PP2A subunit genes. It has been reported that hypomethylation of the PP2A-B55β gene (*PPP2R2B*) promoter induces its expression, which was found to be involved in the expression of estrogen receptors (ERs) in human breast cancer cell lines [38], [39]. Another study demonstrated that the methylation of deoxycytosine in the CpG islands limited the binding of the phosphorylated cAMP response element-binding protein (pCREB) and decreased the activity of the PP2A-Cα (*PPP2CA*) promoter. In contrast, the binding of Sp1 to a GC box within the promoter region was not influenced by DNA methylation [12]. However, for the *PPP2R1A* gene promoter, no evidence was found for the association between DNA methylation and the regulation of gene transcription. We first predicted the presence of concentrated CpG islands in the *PPP2R1A* gene promoter and then speculated that epigenetic mechanisms may be involved in the transcriptional regulation of *PPP2R1A*. The influence of promoter methylation on *PPP2R1A* transcriptional activity was demonstrated using constructs with both unmethylated and methylated forms. Our data indicate that the hypermethylation of the *PPP2R1A* promoter significantly reduced the transcriptional regulatory activity. Furthermore, we used a DNA methylation inhibitor, 5-Aza-dC, in normal hepatocellular L02 cells to confirm the effect of promoter demethylation on gene expression. Our results indicate that the level of *PPP2R1A* mRNA was increased in cells after 5-Aza-dC treatment and that hypomethylation of the promoter was one of the mechanisms responsible for the regulation of *PPP2R1A* expression. Therefore, our results indicate that the methylation status of the *PPP2R1A* promoter might be one of the epigenetic mechanisms regulating key tumor suppressor genes (TSGs) involved in the transcriptional regulatory of the PP2A-Aα gene, which might suggest a novel gene target during hepatocarcinogenesis and could provide a novel route for the development of a new class of chemopreventers and anti-oncological agents. Although the aforementioned results indicate that methylation plays an important role in the regulation of *PPP2R1A* transcriptional activity, the effects of the −241 (−/G) genetic variant on the transcriptional regulation of *PPP2R1A* is not completely dependent on the DNA methylation status present in the promoter region of the gene. A few such instances reported previously in various cancers also support the results of our study and the conclusion that genetic variants/polymorphism and methylation in different target genes can play an independent role in the transcriptional regulatory mechanism [40], [41], [42], [43]. Here, our findings contribute novel information regarding the gene transcription of *PPP2R1A* regulated by polymorphism and methylation in the promoter region through the genetic and epigenetic mechanisms in hepatocarcinogenesis. However, the molecular basis of these association remains to be further determined.

Recently, a few studies have investigated the association between *PPP2R1A* variants and cancer risk. Dupont et al. performed a nested case-control study of Caucasian women to evaluate genetic variations in the intron region of *PPP2R1A* for their potential contribution to the risk of breast cancer. They identified significant risk and protective haplotypes in the PP2A structural subunit Aα isoform (*PPP2R1A*), with an odds ratios (OR) of 1.63 for the risk haplotype (CTCGGCAGGACTCC, 95% CI  = 1.3–2.1) and 0.55 for the protective haplotype (CTAGGCAGGACTCC, 95% CI  = 0.41–0.76). Women with both the *PPP2R1A* risk haplotype and a history of proliferative breast disease had an OR of 2.44 (95% CI  = 1.7–3.5) for the subsequent development of breast cancer [44]. The results of Dupont et al. are consistent with multiple independent lines of study that have all suggested a key role for *PPP2R1A* in the development of cancer, including the viral- and toxin-induced transformation of cell culture systems and the somatic mutation of genes encoding the PP2A subunits in multiple tumor types [45]. Moreover, other studies have demonstrated that some of PP2A subunits play important roles in HCC development or treatment [46], [47], [48]. Duong et al. also reported that Hepatitis C virus-induced overexpression of PP2Ac contributes to hepatocarcinogenesis and inhibits DNA damage repair [49]. However, few studies have investigated the association between *PPP2R1A* promoter polymorphism and HCC genetic susceptibility in diverse ethnic groups. In our case-control study, which was frequency-matched by age (±5 years), sex, we genotyped the −241 (−/G) variant of the *PPP2R1A* promoter to investigate its association with the risk of HCC in a Han Chinese population. The genotype frequencies among controls strongly fit Hardy-Weinberg disequilibrium, suggesting that the selection of subjects was random in this population. Moreover, we observed a protective effect of the −241 (−/G) variant on HCC risk with OR of 0.38 (95% CI  = 0.22–0.67) in −241 (−G)/(GG) combined genotypes in our Han Chinese study population. However, it is not clear how the *PPP2R1A* −241 G allele contributes to the protection against HCC. In our study, we found that the functional −241 (−/G) variant was involved in the transcriptional regulation of the target gene promoter by NF-κB. Recent findings from other researchers have implicated constitutive activation of the transcription factor NF-κB as one of the early events involved in neoplastic progression of the liver. Work from several labs has provided a causal link neoplastic progression through transcriptional regulation of genes involved in cellular transformation, proliferation, survival, invasion and metastasis [50]. The influence of inflammatory signaling on hepatocarcinogenesis can be context dependent, and the deletion of NF-κB-dependent inflammatory responses enhanced the formation of HCC in carcinogen-treated mice [51]. Similarly, the deletion of the NF-κB essential modulator/IκB kinase (NEMO/IKK), an activator of NF-κB, was found to induce steatohepatitis and HCC in mice [52]. In contrast, the inhibition of NF-κB impaired HCC progression in a mouse model of cholestatic hepatitis [53]. The −241 G variant allele in current study significantly decreased the binding affinity of NF-κB in human liver cells compared with the −241 (−) allele variant, which may partially explain the association between the −241 (−/G) variant and HCC risk.

Moreover, in the current study, we found that the protective role of the −241 G variant genotypes was more pronounced among those aged ≤ 40 years old. We speculate that environmental factors, such as a weakening immune system and the overwhelming accumulated exposure to environmental carcinogens in older, male individuals, rather than genetic effects, may account for HCC risk in older subjects [11], [18]. We also observed a significantly lower risk for HCC associated with the *PPP2R1A* variant in females. One possible explanation for this result is that males may be subject to sex-specific differences in exposure to certain risk factors, such as alcohol consumption and smoking cigarettes [11]. Another study indicated that non-environmental endogenous factors could also adversely affect male risk, including higher body mass index (BMI) and higher levels of androgenic hormones [54]. In addition, we found a decreased risk of HCC in cases who were HBV-negative, which is consistent with the findings that the incidence of HCC was significantly lower in immune persons compared with carriers [55]. Several studies have suggested that growth-control genes, such as MAPK, ERK, JNK and others, are involved in HBV-induced hepatocarcinogenesis [56], [57], [58], [59]. NF-κB has been reported to modulate signals in the TNF-α and JNK pathways, as well as affect the role of JNK in TNF-α-mediated apoptosis [15], [60], [61]. Therefore, the −241 (−/G) variant may be more influential in the onset of HCC, although this requires further confirmation. When our previous study and our current findings are taken together, our data suggest that the −241 G variant allele may be a protective factor for HCC via the functional regulation of PP2A-Aα expression by NF-κB signaling.

In summary, our results indicate that the transcription of *PPP2R1A* was regulated by the −241 (−/G) functional variant via the activity of the transcription factor NF-κB targeted to the regulatory promoter region. These findings further improved the functional assays of the −241 (−/G) variant in the promoter region of *PPP2R1A* performed in normal hepatocytes. In addition, our study demonstrates that the transcription of *PPP2R1A* is controlled by the methylation status of the regulatory promoter region and the −241 (−/G) genetic variant. Moreover, the population data provide the first evidence that the heterozygous −241 (−G) genotype of the *PPP2R1A* promoter is significantly associated with the decreased risk of HCC in a southern Chinese Han population when compared with carriers of the −241 (− −) genotype. Our findings provide novel information about the genetic polymorphism and epigenetic methylation in the *PPP2R1A* promoter region and how it alternately influences the transcriptional regulation of PP2A-Aα. In addition, our findings suggest that the −241 (−/G) variant may be an HCC-susceptibility marker in the Chinese population.
